# Radomizing an Antenna for a SAR-Based ETA Radar System While Ensuring Imaging Accuracy: A Focus on Phase Shifts

**DOI:** 10.3390/mi16060720

**Published:** 2025-06-17

**Authors:** María Elena de Cos Gómez, Alicia Flórez Berdasco, Jaime Laviada Martínez, Fernando Las-Heras Andrés

**Affiliations:** TSC, Electrical Engineering Department, University of Oviedo, 33203 Gijón, Spain; florezalicia@uniovi.es (A.F.B.); laviadajaime@uniovi.es (J.L.M.); flasheras@uniovi.es (F.L.-H.A.)

**Keywords:** radome, metasurface, periodic surface, mm-wave antenna, radar antenna, imaging, image quality degradation, synthetic aperture radar (SAR)

## Abstract

The impact of radomization on the radiation pattern of a millimeter-wave antenna for an ETA system utilizing synthetic aperture radar (SAR) is examined with special emphasis placed on the phase shift across both the beamwidth and the bandwidth, rather than the amplitude. Three different radomization approaches, including one based on metasurfaces, are evaluated for a radar antenna operating within the 24.05–24.25 GHz frequency range. Fabricated prototypes, both of the standalone antenna and the radomized version, are tested and compared in terms of electromagnetic image quality. The metasurface-based radome provides the best results among the radomization options analyzed.

## 1. Introduction

Electronic travel aid (ETA) systems designed to assist visually impaired or blind individuals [1] are considered a relevant research topic due to the current number of affected people, the increasing forecasts attributed to the aging population, and the proven impact of visual impairments on life quality [2]. Navigation for visually impaired individuals is challenging due to the insufficient information available for avoiding obstacles and hazards in their path. Without external assistance, orienting and navigating in unfamiliar environments can seem nearly impossible. Although numerous technological solutions have been proposed, none effectively address the daily assistance needs of individuals who are completely blind or visually impaired. In this context, a system capable of providing robust and accurate localization for visually impaired users is essential. White canes and guide dogs are the most widely used aids. The white cane is the simplest, most affordable, and most popular navigation tool. However, it lacks the ability to provide crucial information such as the speed and size of obstacles and the distance to them, which are typically gathered by sight and are essential for perceiving and controlling movement during navigation. Moreover, it does not protect the upper body from potential collisions. Guide dogs, on the other hand, must undergo specialized training. Additionally, there is a necessary adaptation period for both the dog and the individual to become accustomed to each other. Furthermore, guide dogs require extra care and attention to ensure their well-being and effectiveness in assisting their owner. Therefore, alternative or complementary navigating methods are of particular interest as a research topic.

Radar-based ETA systems are found to outperform ultrasonic and optical sensor-based systems, which face limitations in range when operating on highly reflective surfaces and in detecting small openings and have high sensitivity to natural ambient light, respectively [3,4,5].

Radar units typically operate at very high frequencies, which facilitate high resolution due to smaller wavelength, thus allowing for the detection of smaller objects. Additionally, higher frequencies result in more compact radar systems, as the antenna size decreases while maintaining the same gain. The utilization of the Industrial, Scientific, and Medical (ISM) band at 24 GHz (24.05–24.25 GHz) for radar systems offers several notable advantages. This frequency band provides a favorable balance between range and resolution, making it suitable for various applications, including presence detection, motion sensing, and distance measurement. Additionally, the 24 GHz ISM band is characterized by low power consumption and ease of integration into existing systems, which contributes to its cost-effectiveness. For instance, 24 GHz radar systems have been employed in various applications, including industrial control, pedestrian recognition [6], security, collision avoidance [7,8], and biomedical fields [9]. Specifically, for wearable electronic assistance systems, the compact size and lightweight nature of 24 GHz radar modules facilitate seamless integration into wearable devices, ensuring user comfort and unobtrusiveness. Furthermore, the ability to operate effectively in diverse (foggy, smoky, and dusty) environmental conditions underscores the robustness and versatility of 24 GHz radar systems in wearable applications.

While high-range resolution is enjoyed by mm-wave radars, poor angular resolution is suffered in comparison to optical systems. Instead of increasing the number of receiving antennas to enlarge the radar aperture size, synthetic aperture radar (SAR) algorithms are utilized to create a virtual antenna array that is much larger than the physical one [10], achieving higher angular resolution and providing high-resolution images by combining signals from different positions along the radar’s trajectory. Therefore, human movement can be leveraged to extract an electromagnetic image of the surrounding environment [11].

SAR imaging relies on the coherent summation of received signals. Beyond antenna mispositioning [12] and frequency uncertainty [13], the impact of the antenna radiation pattern is recognized as another potential source of image quality degradation. While previous studies have examined the influence of the gain pattern [14], recent research has demonstrated that the phase of the radiation pattern is crucial to the quality of the computed image, particularly regarding phase shifts across the beamwidth and bandwidth [15].

Protecting wearable antennas from potential impacts and inclement weather using radomes is desirable, provided their performance is not degraded. Hence, theoretically, radomes should be electromagnetically transparent. In practice, this implies that radomes must exhibit high transmission and low reflection and absorption within the antenna’s operating band. Metasurfaces can be employed to enhance radome performance or act as radomes themselves [16,17]. For SAR imaging applications, it is essential to consider the potential impact of the radome on the quality of the resulting images, particularly regarding its effect on the phase of the antenna radiation pattern, which has not been analyzed so far.

This research has the potential to significantly advance the field of ETA systems, providing improved mobility and independence for visually impaired individuals, while also contributing to the broader scientific understanding of radar and SAR technologies. The study also highlights the critical role of the phase of the antenna radiation pattern in image quality, an area that has not been extensively analyzed before. Additionally, the investigation into the use of metasurfaces or periodic structures to improve radome performance addresses practical challenges in protecting wearable antennas without degrading their performance.

**Figure 1:**
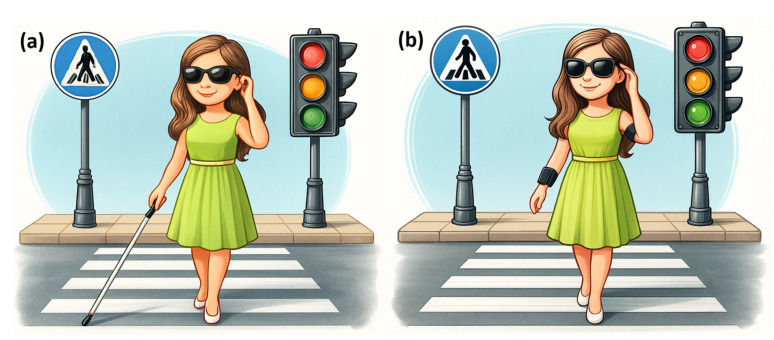
Example of application: current situation vs. SAR-based radar ETA system. (**a**) Visually impaired person using a white cane to avoid obstacles; (**b**) visually impaired person wearing an SAR-based radar ETA system.

Accordingly, the effect of different radomes on the radiation pattern of an antenna for a mm-wave SAR-based ETA system (see Figure 1) is analyzed, with special attention given to potential phase shifts across the beamwidth and bandwidth. Additionally, results of images obtained for the antenna protected by optimized radomes are shown and compared with those corresponding to the un-radomized antenna. Finally, a series of conclusions are drawn.

## 2. Materials and Methods

This section first describes the antenna (ETA radar antenna) that will be used as a reference. Although the antenna itself is an optimized design for this specific application of a short to medium-range blind assistance system, it does not constitute the novelty of this work and should be taken merely as a reference. The novelty lies in studying the influence of potential phase shifts in the radiation pattern across the beamwidth and bandwidth of the antenna, which occur when the reference antenna is protected using different radomes, on the accuracy of electromagnetic imaging. The study carried out is presented immediately after the description of the reference antenna.

### 2.1. ETA Radar Antenna

Microstrip grid array antennas offer several advantages, especially in radar applications within millimeter-wave frequency bands. These antennas combine the benefits of grid array structures (high radiating element density while minimizing the number of feeding lines external to the antenna structure) [18] with the compact and planar nature of microstrip technology [19], showing additional advantages such as increased gain, cross-polarization control, and wider bandwidth compared to conventional patch radiators. This integration results in medium gain and narrow beamwidth, which are essential for precise target detection and tracking [20]. The microstrip design also allows for easier integration with other circuit components, enhancing overall system efficiency [21]. Additionally, these antennas exhibit low-profile and lightweight characteristics, making them suitable for various applications, including airborne and spaceborne radar systems [22]. Their ability to operate efficiently at high frequencies further underscores their suitability for modern radar applications where precision and reliability are paramount [21,23].

In a grid array antenna (GAA), the number of loops determines directivity and functional bandwidth. Increasing the number of loops enhances directivity but reduces functional bandwidth. Taking into account the trade-off between the range and coverage area of any radar system and according to the recent literature [12,24,25], for the target application, antennas with a beamwidth of about 30–40 degrees are required if the blind user is to be forewarned of obstacles in the short to medium range (up to 2 m). Eco-friendly polypropylene (PP), with relative dielectric permittivity ε_r_ = 2.2 and loss tangent tan δ = 0.002, has proven to be suitable as an antenna substrate at 24 GHz [26], and when cladded with aluminum (Al), which is also eco-friendly, it results in devices that contribute to sustainable development and the circular economy [27,28].

**Figure 2:**
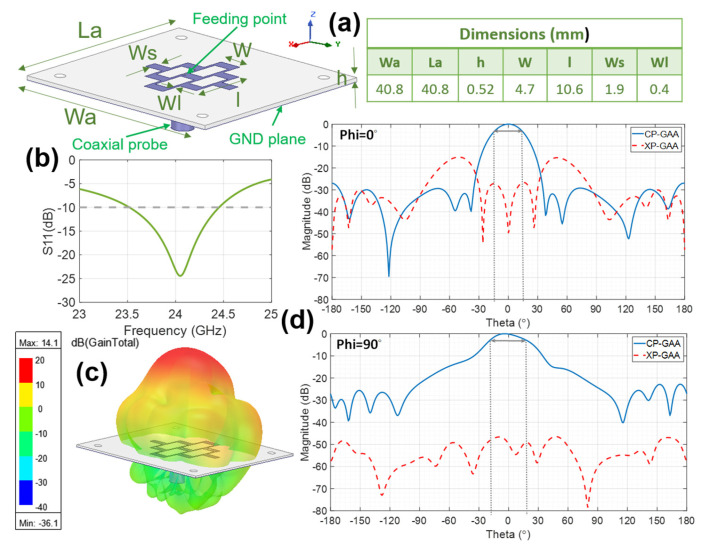
Reference ETA radar antenna: (**a**) grid array antenna geometry with visible metallic parts and optimized dimensions; (**b**) S_11_ (dB); (**c**) three-dimensional radiation pattern at 24.15 GHz; and (**d**) co-polar (CP) and cross-polar (XP) components of the normalized radiation patterns cuts for Phi = 0° and Phi = 90° at 24.15 GHz, with the half-power beamwidth (HPBW) indicated in dotted lines.

In [28], the design of an antenna for a medium to long-range ETA radar system is shown. It is a considerably directive antenna with a narrow beamwidth in both Phi = 0° and Phi = 90° cuts of the radiation pattern, which requires a high number of rectangular loops in the radiating grid (32) and, consequently, a high number of radiating elements (41). The design process, eco-friendly materials’ characterization, and prototype manufacturing procedure are explained in [28], as well as the challenges involved due to the employment of materials not commonly used for antennas. In the present work, to achieve the target beamwidth (about 30–40 degrees) for a short to medium-range system, the number of loops and, therefore, the number of radiating elements in the microstrip GAA are reduced accordingly in such a way that (with the help of 3D commercial electromagnetic simulation software based on FEM) a radiating grid composed of four rectangular loops with nine radiating elements is achieved. This aluminum radiating grid, situated on the polypropylene substrate and fed with a 50 Ω coaxial probe near its center, is represented in Figure 2a, along with its dimensions. The polypropylene substrate is backed by an aluminum ground plane. Four holes of 2 mm diameter also appear near the corners of the dielectric to secure the antenna in the measurement setup. The guided wavelength λg at the central frequency of the band (24.15 GHz) on the PP material is 9.85 mm. Initial values for W and l are set to achieve antenna resonance at this frequency (W = λg/2, l = λg), while Ws and Wl are initially chosen based on manufacturing constraints. Using iterative parametric sweeps in simulation, W and l are adjusted to define the band. Once this is accomplished, Ws and Wl are refined to optimize the radiation pattern, taking into account that Ws governs the width of the aperture and the side lobe level (SLL), whereas Wl controls the radiation level of cross-polar components.

As can be observed in Figure 2b, the antenna exhibits close to 4% impedance matching bandwidth (S11 below −10 dB), running from 23.51 GHz to 24.45 GHz, which is considerable, with remarkable matching levels within the target band (24.05–24.25 GHz).

It is also worth highlighting that the radiation characteristics of the antenna (peak realized gain (G), peak directivity (D), radiation efficiency (η), and front-to-back ratio (FTBR)) are optimal for the intended application across the entire target frequency band: G (≥13.7 dBi), D (≥14.2 dB), η ≥ 89%, and FTBR ≥ 22.9 dB. Focusing specifically on the results at the center of the band (24.15 GHz), the corresponding values are G = 14 dBi, D = 14.3 dB, η = 93% and FTBR = 22.9 dB. It should be considered that η and FTBR are crucial in wearable applications, and the results provided by this antenna are very suitable for such devices and for the intended use according to the desired application. From the radiation pattern cuts for Phi = 0° and Phi = 90° (see Figure 2d), broadside radiation with a low side lobe level (SLL) and half-power beam width (HPBW) of ≥ 30° is obtained, which is suitable for the desired application. Moreover, the GAA exhibits a broad 3 dB gain–drop bandwidth (11%).

### 2.2. Radomized ETA Radar Antenna

**Figure 3:**
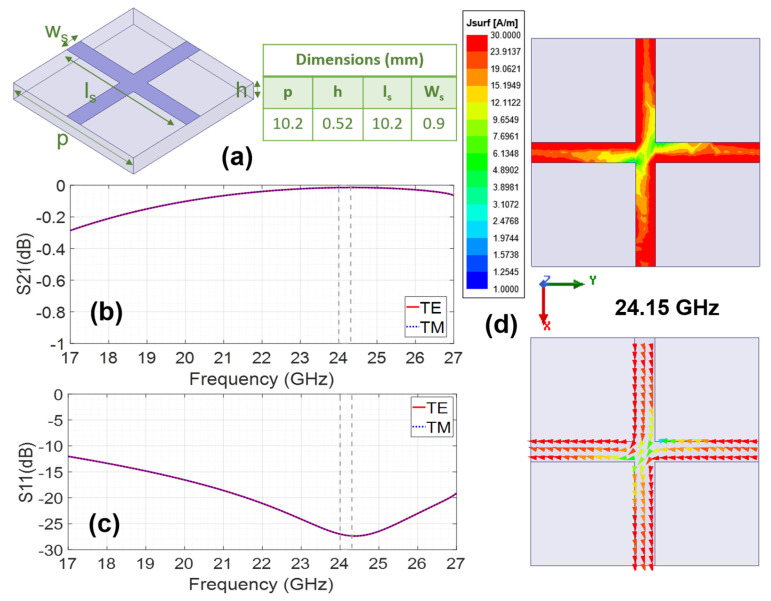
Al-PP based metaradome: (**a**) geometry of the unit cell and optimized dimensions; (**b**) transmission properties (S_21_ (dB)); (**c**) reflection properties (S_11_ (dB)); (**d**) surface current distribution on the unit cell at 24.15 GHz.

Among the fundamental styles of conventional radomes, categorized by dielectric wall construction, there are (1) monolithic half-wave solid walls, (2) thin-wall monolithic radomes (with thickness ≤ 0.1 λg at the highest operating frequency), and (3) sandwiched multi-layered walls that expand the operating bandwidth. The latter are unsuitable for the intended application due to either the required thickness, which would make the device heavier and uncomfortable, or the complexity and higher implementation cost. A monolithic half-wave wall radome on PP operating at 24 GHz should be 4.1 mm thick and positioned at least 6.21 mm from the antenna. This option is not convenient at all as it results in a bulkier, heavier, less discreet, and less comfortable antenna. Nevertheless, its impact on the radiation pattern will also be studied for comparison. Conversely, a thin monolithic radome, requiring a thickness ≤ 0.83 mm if PP-based, appears to be a more suitable solution. Finally, a third approach involves utilizing the properties of metasurfaces or periodic surfaces to design metaradomes or metadomes as an alternative to conventional radomes. The unit cell geometry of a PP-based metaradome (MTR) designed for operation at the target frequency band is shown in Figure 3a, along with its optimized dimensions.

When designing the geometry of the unit cell metallization, it is essential to consider the equivalent circuit in terms of inductive (L) and capacitive (C) elements, so that the resonance frequency is as desired, covering at least the necessary bandwidth and being sufficiently stable under oblique incidence conditions for both TE and TM polarized incident plane waves. This design task involves a certain component of prior experience (derived both from the study of many periodic surface designs available in the literature for various uses and from previously published own designs) [29,30,31], knowledge of the equivalent circuits of metal–dielectric structures [32,33], and one’s own intuition and creativity. It must be kept in mind that when working at such very high frequencies, only simple geometries should be considered, so that they can be manufactured using conventional microwave-printed circuit techniques to avoid increasing costs. With all this in mind, a cross-shaped geometry has been chosen, whose arms exhibit inductive behavior (the longer (increasing ls) and narrower (decreasing Ws) they are, the higher the inductive value), while the gaps between them, made of dielectric material, exhibit capacitive behavior (the smaller the space between the arms of the cross, and the wider the metal strips, the higher the equivalent capacitance). The resonance frequency, given that this geometry corresponds to a parallel LC equivalent circuit, is inversely proportional to the square root of the product of the equivalent L and C. Furthermore, if the dielectric thickness is reduced, the resonance frequency increases, and its performance as a radome improves because transmission enhances (S_21_ increases) and reflection reduces (S_11_ decreases). In this case, the thickness used is the one available for polypropylene h = 0.52 mm, so the rest of the dimensions have been optimized through electromagnetic simulation using finite element method (FEM) commercial software, the same used for the design of the reference antenna, performing parametric sweeps until the desired behavior is achieved, thereby determining the optimal dimensions.

From Figure 3b,c, it can be observed that maximum transmission and minimum reflection are achieved at the GAA operating band. Accordingly, a high surface current level is observed at the center frequency (24.15 GHz), as shown in Figure 3d. Additionally, the angular stability of the MTR, in terms of both transmission and reflection coefficients, has also been analyzed since it is crucial for a radome. In the 24.05–24.25 GHz band, the MTR is fully stable under oblique incident TE-polarized plane waves (up to θ = 48°) and highly stable for TM-polarized plane waves (up to θ = 26°).

**Figure 4:**
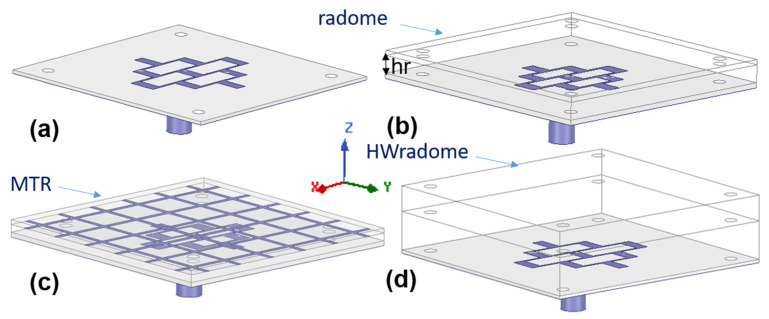
ETA radar antenna and radomization approaches under study: (**a**) reference antenna, GAA, (**b**) GAA+radome, (**c**) GAA+MTR, and (**d**) GAA+HW-radome.

The reference antenna, GAA, and the three PP-based approaches considered for its radomization are as follows: a thin-wall monolithic radome, hereafter named radome, and a metaradome (MTR) and monolithic half-wave wall, hereafter named HW-radome. These are shown in Figure 4. Both the radome and the MTR have a thickness of h = 0.52 mm.

#### 2.2.1. Radiation Pattern Magnitude

**Figure 5:**
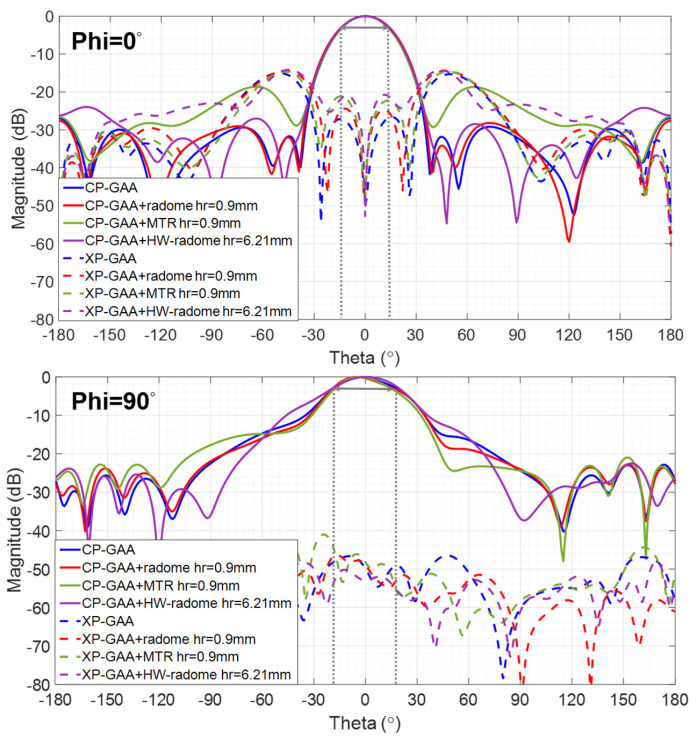
Magnitude of co-polar (CP) and cross-polar (XP) components for Phi = 0° and Phi = 90° radiation pattern cuts at 24.15 GHz corresponding to the GAA and the three radomization options under analysis.

The influence of the distance from the GAA to the considered radomes, hr, in terms of the radiation pattern magnitude (see Figure 5) and radiation properties (see Table 1) is analyzed. The HW-radome has to be arranged at hr = 6.21 mm to work properly, so other distances are not considered for this approach. According to simulations, it would provide G = 12.9 dBi, η = 88% and FTBR = 25.7 dB at 24.15 GHz, which compared to GAA (the reference antenna) means a slight reduction in G (1 dB) and a significant reduction in η (5%), along with a 3 dB improvement in FTBR, all with a substantial increase in thickness, volume and weight.

According to Table 1, the MTR can be arranged much closer to the GAA reference antenna than the radome (hr = 0.7 mm vs. hr = 1.4 mm) for identical η and similar G and FTBR. It is remarkable that at hr = 0.9 mm, the MTR provides 5% higher η for similar G and FTBR. At such a distance (hr = 0.9 mm) and the one required for the HW-radome (hr = 6.21 mm), the HPBW = 30° for Phi = 0° and HPBW = 40° for Phi = 90° pattern cuts are well preserved for both radomes and the MTR (see Figure 5). The HW-radome slightly widens the −10 dB beamwidth for the Phi = 90° cut, whereas the MTR slightly increases SLL for the Phi = 90° cut (but keeping it ≤−18 dB), which is suitable for the intended ETA application.

#### 2.2.2. Radiation Pattern Phase

**Figure 6:**
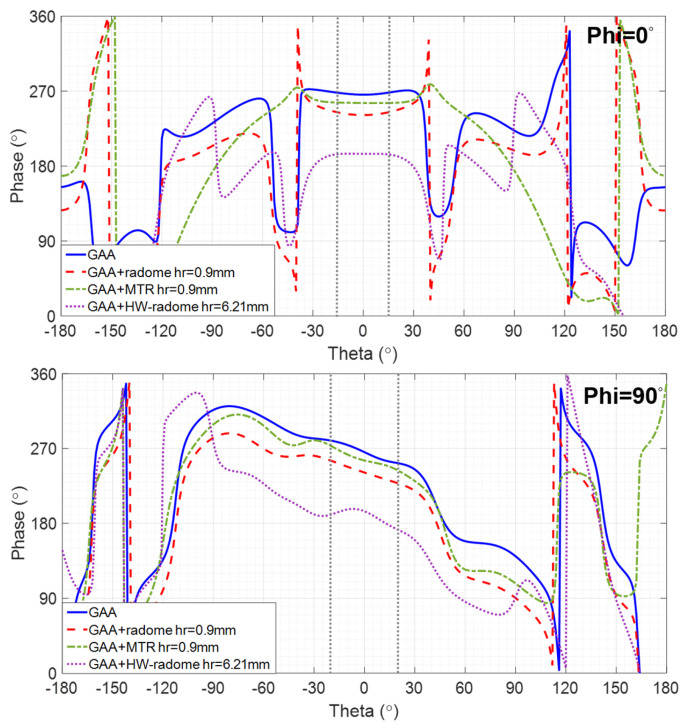
Phase of the CP component for Phi = 0° and Phi = 90° radiation pattern cuts at 24.15 GHz corresponding to the GAA and the three radomization options under analysis.

The phase variation across the beamwidth for the co-polar (CP) component (the cross-polar (XP) component is ignored due to its low relevance) is shown in Figure 6. For Phi = 0°, it is remarkable that the MTR exhibits a constant phase, which is desirable for high image quality. The GAA and both radomes feature a small phase shift, with a slightly curved feature, which is more noticeable for the radome.

For Phi = 90°, the GAA, the radome, and the MTR exhibit an almost linear phase shift, with a slope of −0.7 that, according to [34], would lead to a target displacement on the image smaller than λ. However, the phase shift of the HW-radome follows a quadratic characteristic, which will result in image distortion.

**Figure 7:**
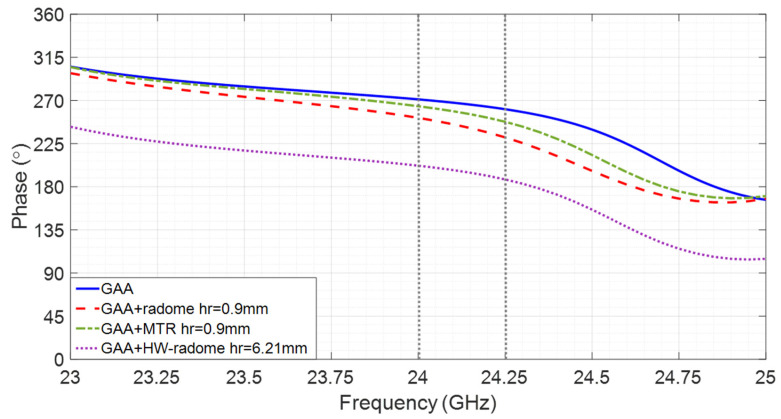
Phase shift vs. frequency of the GAA and the three considered radomization options for Theta = 0°.

Regarding the phase shift across the bandwidth for theta = 0°, a linear characteristic with a negative slope can be observed in Figure 7 either for the GAA or the radomized cases, considering a range of 24.05–24.25 GHz. Such a phase term can be expressed as e−^jkL^, with k being the wavenumber. L is usually estimated during an initial calibration stage and removed by multiplying by the opposite phase [34] to obtain a constant phase over the operating frequency band.

For a wider bandwidth (e.g., in case the resolution in the range needs to be improved) from 23 GHz to 25 GHz, in addition to the linear term, the phase displays a certain ripple that cannot be compensated for by linear calibration and that results in a reduction of the dynamic range for images parallel to the acquisition plane [15]. As the ripple does not fit a pure sinusoidal feature, as characterized in [15], its standard deviation σ is used to quantify its variation. It can be observed in Table 2 that the radomization reduces the phase ripple of the GAA. Furthermore, the MTR behavior is very similar to the HW-radome while being much thinner, lighter, and arranged much closer. As could be expected, the standard deviation is almost null in the range of 24.05–24.25 GHz for all cases, since there is no ripple.

**Figure 8:**
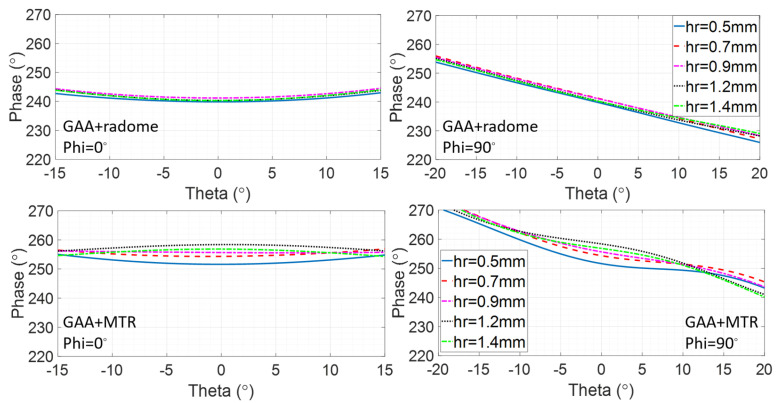
Phase shift across the beamwidth for Phi = 0° and Phi = 90° at 24.15 GHz for the GAA+radome and the GAA+MTR at several hr.

Furthermore, the influence of the distance from the GAA to the considered radomes, hr, in terms of the radiation pattern phase has been also analyzed. The HW-radome should be kept at hr = 6.21 mm, and therefore it is the radome and the MTR that are taken into account. It can be observed in Figure 8 that the phase shift for the hr variation from 0.5 mm to 1.4 mm is very small in both cases. For Phi = 0°, the radome phase shift exhibits a slightly curved feature, while showing a linear one with a −0.7 slope across the beamwidth for Phi = 90° for all the hr values. The GAA+MTR at hr = 0.9 mm shows a constant phase for Phi = 0°, with very slight curved variations around it for the other hr values and an almost linear phase shift for Phi = 90°; it also exhibits small variations for the considered increasing and decreasing hr values. Hence, hr = 0.9 mm seems to be the most suitable value to be used in practice.

### 2.3. Point Spread Function (PSF)

The point spread function (PSF) is a critical concept in optical imaging systems, representing the response of an imaging system to a point source of light. It essentially characterizes how a point source is imaged by the system, detailing the distribution of light in the resultant image. The PSF can be derived through the convolution of the actual image with the system’s impulse response. In linear and space-invariant systems, the PSF is obtained by computing the inverse Fourier transform of the optical transfer function (OTF). Experimentally, the PSF can be measured by imaging a point source and recording the resultant light distribution. The PSF is indispensable for assessing the performance of imaging systems. It quantifies the extent of blurring or spreading that an image undergoes due to the system’s imperfections. This function is pivotal in fields such as astronomy, microscopy, medical imaging, and photography, where it aids in enhancing image resolution and fidelity.

In the same manner, the PSF is a fundamental tool in radar imaging systems, particularly in synthetic aperture radar (SAR) applications. It characterizes the system’s response to a point source, providing insights into the resolution and quality of the radar images. The PSF is crucial for understanding the degree of blurring and the spatial resolution of the radar system. It serves as a diagnostic tool to compare radomes in an SAR-based ETA radar system by determining which radome offers superior electromagnetic image quality. To this end, the following methodology is employed.

**Figure 9:**
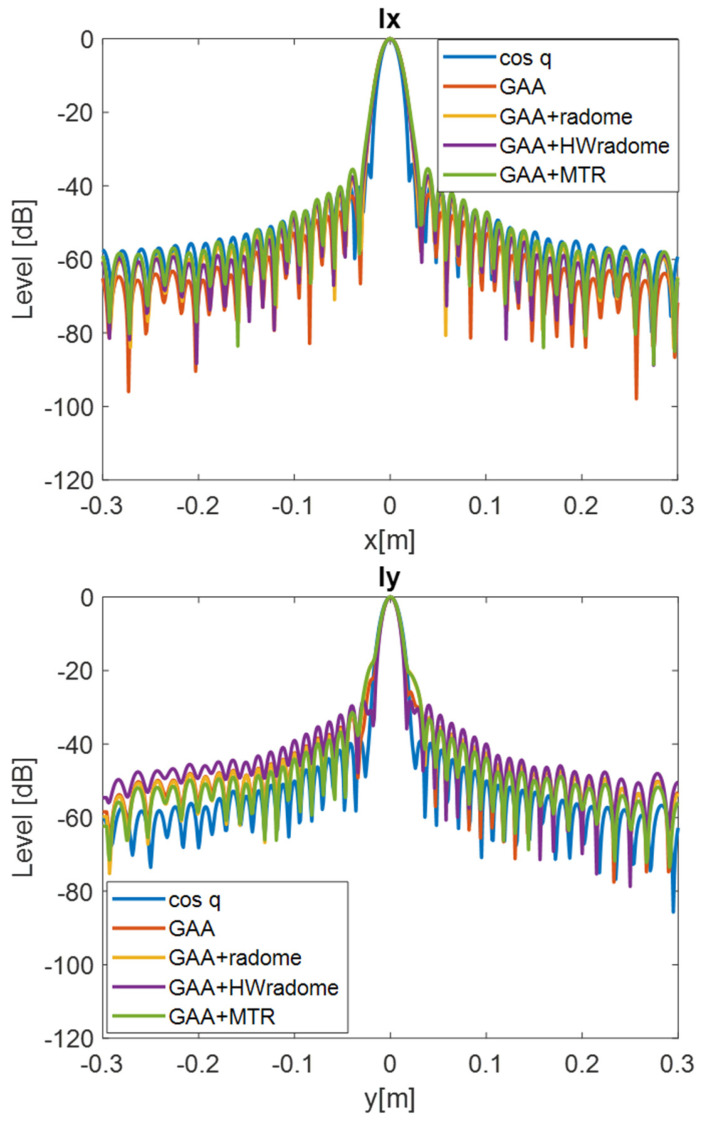
PSF obtained for the ideal Cos q radiation pattern, the un-radomized GAA, the GAA + radome, GAA+HWradome, and GAA+MTR for the 24.05–24.25 GHz band in the *X*-*Y* plane.

**Figure 10:**
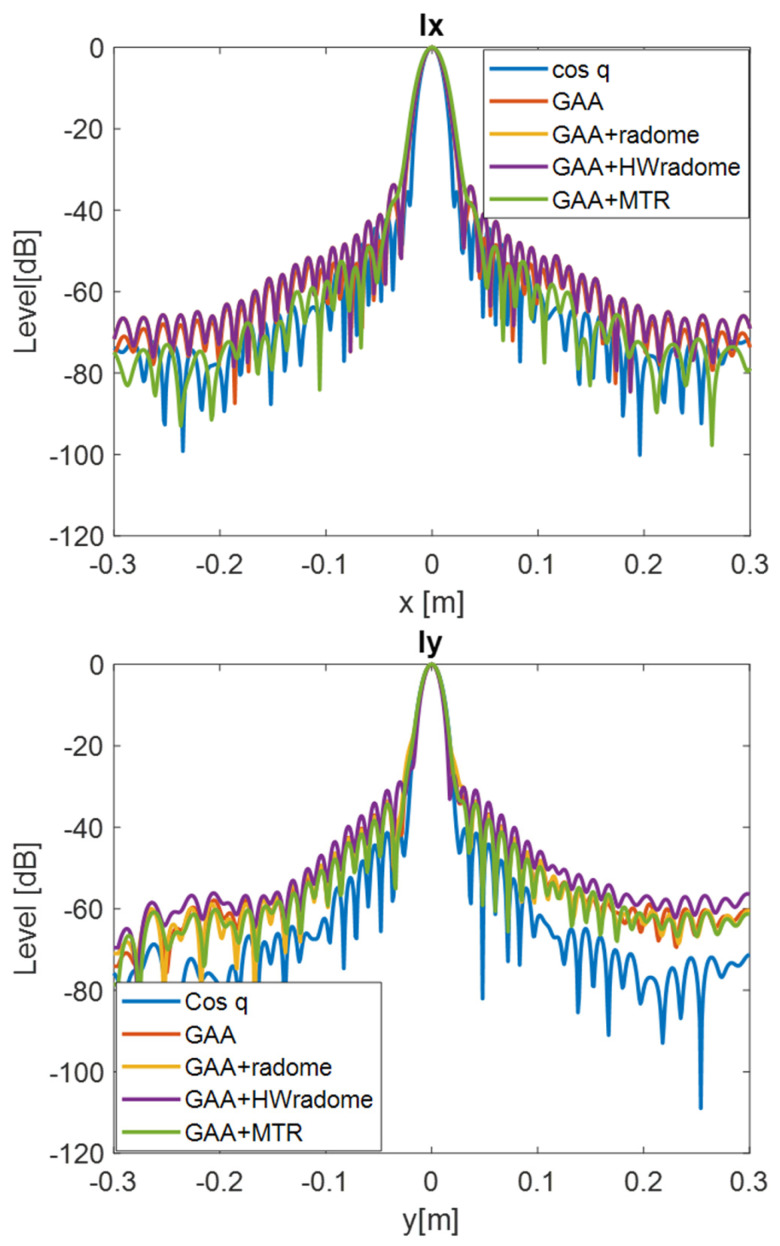
PSF obtained for the ideal Cos q radiation pattern, the un-radomized GAA, the GAA + radome, GAA+HWradome, and GAA+MTR for the 22–26 GHz band in the *X*-*Y* plane.

**Figure 11:**
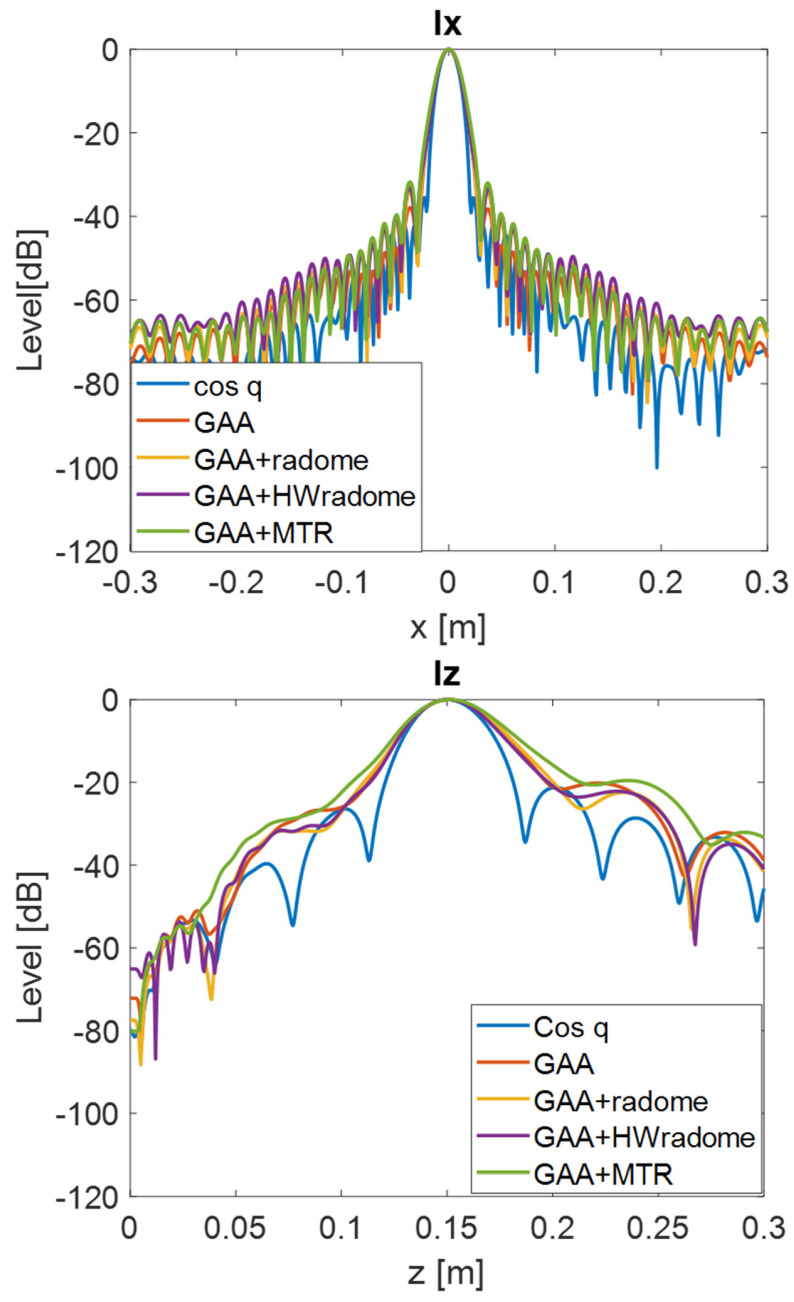
PSF obtained for the ideal Cos q radiation pattern, the un-radomized GAA, the GAA + radome, GAA+HWradome, and GAA+MTR for the 22–26 GHz band in the *X*-*Z* plane.

**Figure 12:**
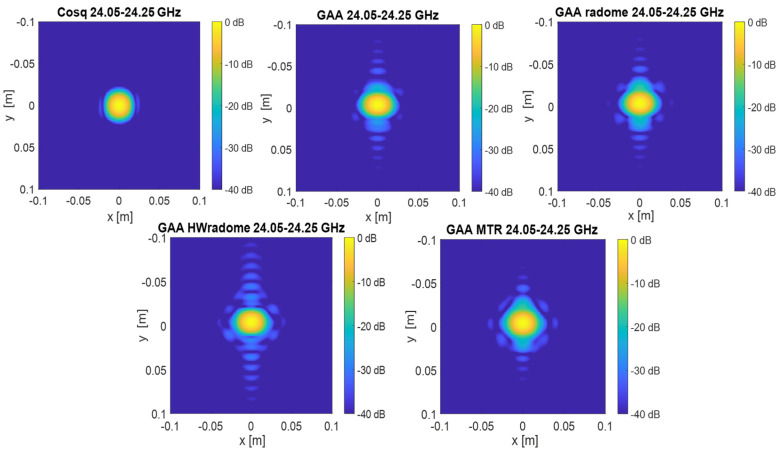
Two-dimensional electromagnetic images of the point source in the *X*-*Y* plane obtained with the Cosq, the un-radomized GAA, GAA+radome, GAA+HWradome, and GAA+MTR for the 24.05–24.25 GHz frequency band.

**Figure 13:**
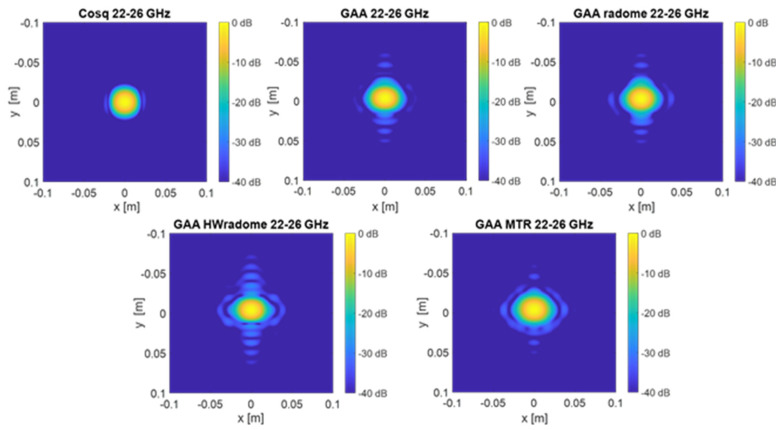
Two-dimensional electromagnetic images of the point source in the *X*-*Y* plane obtained with the Cosq, the un-radomized GAA, GAA+radome, GAA+HWradome, and GAA+MTR for the 22–26 GHz frequency band.

1. PSF retrieval, which is achieved by positioning a point source in front of the randomized antenna and obtaining the field distribution in the resultant image. This procedure is repeated for each radome under consideration. An icosahedron is often employed as a point source in radar imaging due to its geometric properties. The icosahedron, with its 20 equilateral triangular faces, approximates a spherical shape, providing a uniform radar cross-section (RCS) from multiple viewing angles. This uniformity is essential for calibration purposes, ensuring consistent and reliable measurements across different orientations. The use of an icosahedron helps in minimizing the effects of directional biases and provides a stable reference for evaluating the radar system’s performance [35]. In order to be electrically small, the diameter of the icosahedron is set to λ/10. Figure 9 and Figure 10 show the 2D PSF (*X* and *Y* axes) obtained for a cosine-q (cosq) reference radiation pattern (with q = 6, so that the directivity equals the one of the GAA un-radomized antenna), for the un-radomized GAA antenna, and for the three radomes under analysis, both for the 24.05–24.25 GHz frequency band and for an extended 22–26 GHz band to ensure range resolution.
2. PSF analysis, which is performed by comparing the PSF obtained for each radome. To this aim, the field distribution and the degree of blurring are examined. A radome that induces less spreading will exhibit a more concentrated PSF, thereby enhancing image quality. The 3 dB bandwidth is typically measured between the points where the amplitude of the PSF falls to half of its maximum value (i.e., −3 dB points). The secondary lobe level refers to the amplitude of the side lobes relative to the main lobe. In an ideal cosine-q pattern, the secondary lobes are the smaller peaks that appear on either side of the main lobe. The level of these secondary lobes is usually expressed in decibels (dB) relative to the peak of the main lobe. The secondary lobe level is important for understanding the amount of energy that is spread outside the main lobe, which can affect the resolution and contrast of the imaging system. From Figure 9, it can be observed that the PSF varies very little in the two cuts (*X* and *Y* axes) and is very similar for all the cases under study. The same applies for Figure 10, in which the extended 22–26 GHz frequency band is considered. In accordance with these statements, from Table 3 and Table 4, it can be observed that the −3 dB bandwidth of the PSF is almost identical for all the considered devices and very similar to the one of the cosq pattern whether in the 24.05–24.25 GHz or in the 22–26 GHz band. In the *X* axis, the −3 dB bandwidth is slightly narrower for the GAA+MTR than for un-radomized GAA and the other randomization approaches for both frequency bands. Considering the un-radomized antenna and the radomization approaches, the secondary lobe level is slightly lower in the *X* axis than in the *Y* one for the 24.05–24.25 GHz. For the extended frequency band, the difference is less remarkable, and the GAA+MTR exhibits similar level for both axes. In all cases and for both frequency bands, the secondary lobe levels are very low (below −30 dB) but for the HW-radome in the *Y* axis, which is slightly higher; therefore, good resolution and contrast would be expected for the PSF images.
  Considering the PSF in the *X*-*Z* plane shown in Figure 11 and as could be expected for the *Z* axis, in all cases, the main lobe is centered at 15 cm, which matches the target position. In the *X* axis, once again, the secondary lobe level is well below −30 dB for all the configurations, and the −3 dB bandwidth is very similar for all of them (see Table 5).

3. Image quality assessment, which is carried out by utilizing the PSF to evaluate the spatial resolution and contrast of the images produced with each radome. PSF images for the *X*-*Y* cuts of reflectivity over the plane of the point-like target were first obtained [15]. From the images depicted in Figure 12 and Figure 13, it can be observed that the point-like target is perfectly detected in all the considered situations and for both frequency bands. In agreement with the PSF results, the effect of the secondary lobe level on the image quality is lower for the *X*-axis; meanwhile, for the *Y*-axis, the worst case is for the HWradome, exhibiting higher blurring as could be expected from Table 3 and Table 4. On the *Y*-axis, such an effect is lower for the MTR. On the *X*-axis, the effect starts to be noticeable around the target for the GAA without a radome and then increases slightly, being very similar for all radomes.

**Figure 14:**
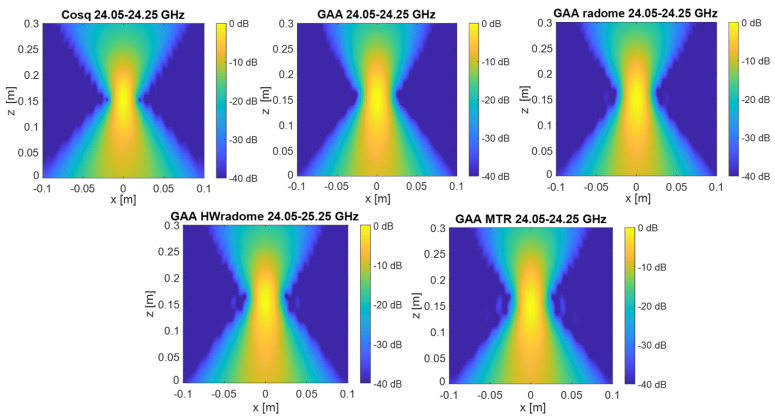
Two-dimensional electromagnetic images of the point source in the *X*-*Z* plane obtained with the Cosq, the un-radomized GAA, GAA+radome, GAA+HWradome, and GAA+MTR for the 24.05–24.25 GHz frequency band.

**Figure 15:**
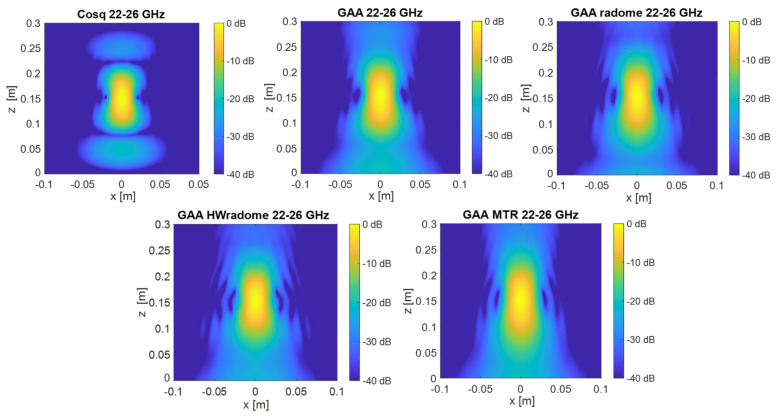
Two-dimensional electromagnetic images of the point source in the *X*-*Z* plane obtained with the Cosq, the un-radomized GAA, GAA+radome, GAA+HWradome, and GAA+MTR for the 22–26 GHz frequency band.

Regarding the images of the point-like target obtained in the *X*-*Z* plane, for the 24.05–24.25 GHz range (see Figure 14), the images are very similar to each other, with the effect of secondary lobes being slightly noticeable for the three radomes considered. When the 22–26 GHz band is considered (see Figure 15), the point-like target image is better defined in all cases. The radomization does not degrade the image quality.

## 3. Results

Prototypes of the GAA reference antenna and the MTR on Al-cladded PP were fabricated with LPKF ProtoMat milling machine (LPKF, Garbsen, Germany). Four holes were drilled at the corners to fix and align the GAA to either the radome or the MTR. In addition, a PP-based radome and a 0.9 mm thick foam slab to be used as separator were cut and drilled (see Figure 13).

### 3.1. Imaging Results

#### Electromagnetic Images

**Figure 16:**
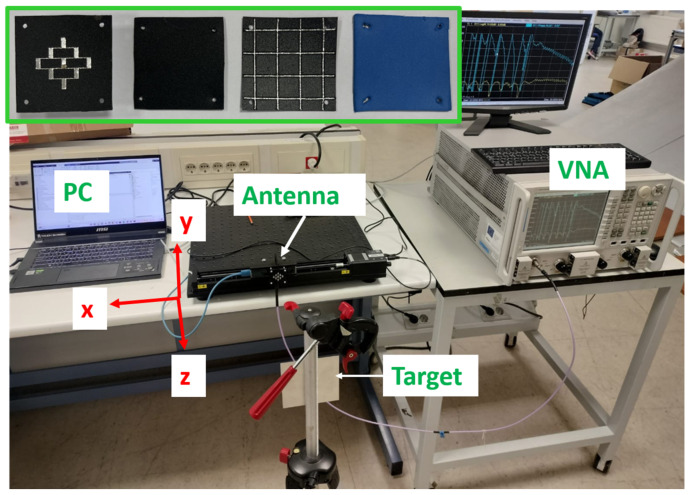
Prototypes of the GAA, radome, and MTR and measurement setup.

A pure monostatic measurement setup was used for the imaging as it is preferable when studying the effects of a type of antenna (so that a single antenna was used as both transmitter (Tx) and receiver (Rx)). The target was a squared metallic plate with dimensions 10 cm × 10 cm, and it was located 15 cm in front of the antenna. Taking into account that the widest beamwidth of the considered antennas (either GAA or radomized GAA with radome or MTR) was ±20°, this distance still ensured that only the target was illuminated, avoiding reflections from other objects as the footprint was approximately 11 cm. The target was kept static, while the antenna under test was moved along a 10 cm path in the *x*-axis by means of a motorized linear micropositioner (see Figure 16). This linear movement was consistent with short-term trajectories, which can be approximated as linear, since long-term acquisition would suffer from cumulative positioning errors [11]. Moreover, it also helped to avoid introducing additional artifacts in the image due to irregular trajectories, which would hinder the actual impact of the radomes.

Aiming to achieve a suitable range resolution to distinguish the target and its range position, measurement data of S_11_ in an extended frequency band (22–26 GHz) were acquired with a step of 2 mm (∼λ/4 at the highest frequency) to satisfy the sampling conditions of the Nyquist theorem. Calibrations and correction of the L term were performed.

**Figure 17:**
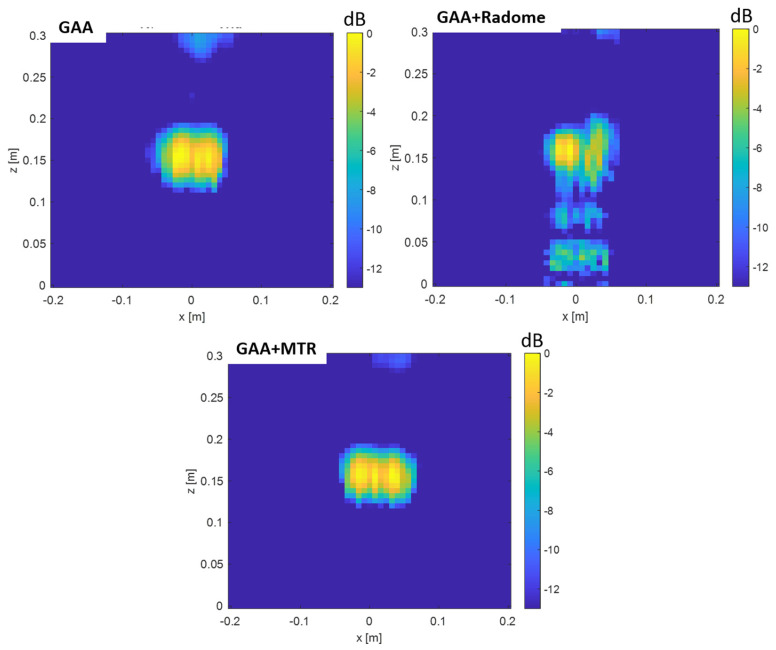
Electromagnetic images obtained with the GAA, GAA+radome, and GAA+MTR for the flat plate.

**Figure 18:**
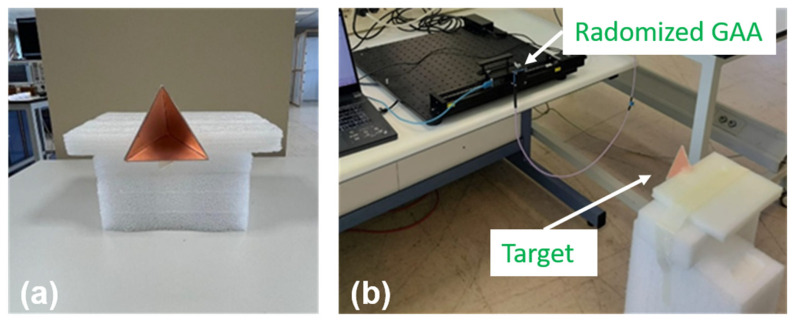
Trihedral corner reflector: (**a**) target element, (**b**) setup at 50 cm.

In addition to the flat plate results (see Figure 17), the analysis of a trihedral corner reflector, whose outer edges size are 5 cm, was also considered (see Figure 18). In this case, the distance was set to 50 cm. The target was placed on a foam bed to avoid any reflection, and the same steps and calibration approach were followed.

**Figure 19:**
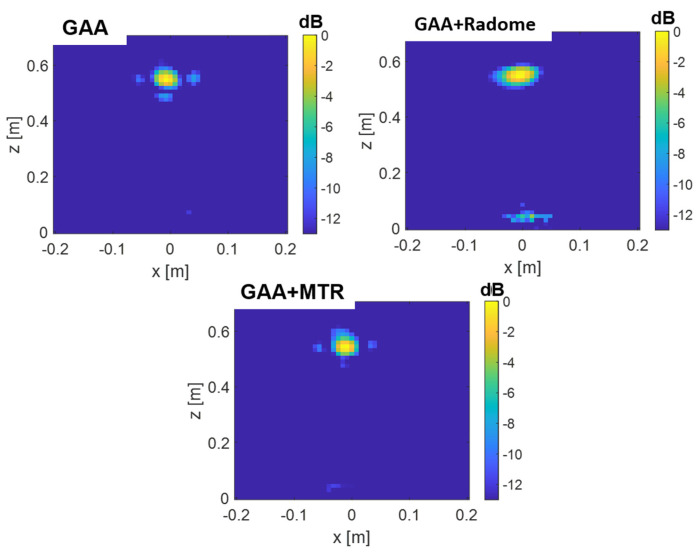
Electromagnetic images obtained with the GAA, GAA+radome, and GAA+MTR for the corner reflector case.

The results for this target are shown in Figure 19 for the following cases: GAA, GAA with radome, and GAA with MTR. These results are consistent with those obtained for the plate, as the outcomes for GAA and GAA+MTR are similar. However, the latter benefits from the protective layer of the radome, while the GAA with radome shows poorer performance, exhibiting a wider lateral resolution.

## 4. Discussion

From Figure 17, it can be observed that the GAA antenna alone provides an electromagnetic image of the target that properly fits its size, with a very slight shift in the *z*-axis, since the target is detected at 15 cm from the antenna, as expected. This is consistent on the one hand with the absence of deformation in the width of the PSF in the *X*-axis (where there is resolution in measurement) when the antenna is radomized and, on the other hand, with the level of the *Z*-axis PSF at the target position. Moreover, the same applies for the GAA+MTR, with minor distortion but with the advantage of protecting the antenna. In fact, the GAA+MTR exhibits a lower artifact in the upper part if compared with the GAA antenna itself. However, the GAA+radome generates the target image with some distortion and artifacts in the lower part. This can be attributed to some extent to the slightly curved characteristic exhibited by its phase across the beamwidth for the Phi = 0° cut. Furthermore, as previously mentioned, on the *X*-axis, the −3 dB PSF bandwidth is slightly narrower for the GAA+MTR than for the un-radomized GAA and the other randomization approaches for both frequency bands. Moreover, in addition to the aforementioned points, it is important to consider that, in practice, any minor misalignment or variation in the placement of the radome on the antenna can result in reflections within the radome material itself, leading to artifacts. Conversely, the MTR has been specifically designed to avoid reflecting signals (across a wide angular range) within a band that comfortably encompasses the one used in measurements. Therefore, it is clear that the MTR outperforms the radome in terms of electromagnetic imaging accuracy while requiring the same device size and total profile.

All these results show that the study of the radiation pattern phase along with the analysis of the PSF and the point target images obtained in simulation truly allow prediction of the image quality that will be achieved before manufacturing the prototypes, which is especially useful. That said, there are obviously some factors that occur in practice (such as manufacturing tolerances and misalignments in prototype assembly and/or radome placement, among others) that cannot be predicted during the simulation stages.

Future research directions could, for example, explore the potential design of metaradomes that not only preserve but, if possible, even improve the image quality achieved.

## 5. Conclusions

The impact of various radomes on the radiation pattern of an antenna within a millimeter-wave SAR-based ETA system has been examined, focusing not only on magnitude but also on potential phase shifts across both beamwidth and bandwidth.

The radome and the MTR offer a significantly thinner, lighter, and more comfortable fully operational prototype compared to the HW-radome, with superior performance regarding phase shifts across beamwidth and bandwidth. The MTR surpasses the radome in terms of radiation efficiency when positioned at the same distance from the antenna and demonstrates enhanced performance at shorter distances.

It has been demonstrated that the GAA can be radomized while preserving imaging accuracy, with the MTR emerging as the optimal choice among the options considered.

## Figures and Tables

**Table 1:** Radiation properties vs. height from the GAA reference antenna.

	GAA + Radome			MTR-GAA		
hr (mm)	G (dBi)	η (%)	FTBR (dB)	G (dBi)	η (%)	FTBR (dB)
0.5	12.8	77	24.4	13.1	87	21.5
0.7	12.3	81	23.5	13.4	88	22.7
0.9	13.4	83	23.8	13.6	88	24.7
1.2	13.7	86	23.9	13.8	88	25.1
1.4	13.8	88	24.5	13.6	87	25

**Table 2:** Phase shift across the bandwidth for Theta = 0°.

	[24.05–24.25] GHz		[23–25] GHz	
	L	σ	L	σ
GAA	−42.8	0.17	−61.2	14.37
GAA+radome	−86.0	0.51	−73.2	10.14
GAA+MTR	−68.6	0.47	−71.4	12.88
GAA+HW-radome	−60.7	0.42	−69.4	13.25

**Table 3:** Details of the −3 dB PSF bandwidth and secondary lobe level for the *X* and *Y* axes in the 24.05–24.25 GHz range.

IX	Cos q	GAA	GAA+radome	GAA+HWradome	GAA+MTR
−3 dB PSF BW (m)	0.0142	0.0164	0.0163	0.0159	0.0149
PSF SLL (dB)	−34.2	−42.3	−38.6	−37.5	−35.4
**IY**	**Cos q**	**GAA**	**GAA+radome**	**GAA+HWradome**	**GAA+MTR**
−3 dB PSF BW (m)	0.0146	0.0132	0.0134	0.0128	0.0138
PSF SLL (dB)	−39.2	−32.2	−31.8	−28.5	−31.5

**Table 4:** Details of the −3 dB PSF bandwidth and secondary lobe level for the *X* and *Y* axes in the 22–26 GHz range.

IX	Cos q	GAA	GAA+radome	GAA+HWradome	GAA+MTR
−3 dB PSF BW (m)	0.0143	0.0160	0.0156	0.0152	0.0149
PSF SLL (dB)	−35.6	−38.9	−34.4	−34.0	−32.0
**IY**	**Cos q**	**GAA**	**GAA+radome**	**GAA+HWradome**	**GAA+MTR**
−3 dB PSF BW (m)	0.0147	0.0137	0.0140	0.0131	0.0145
PSF SLL (dB)	−40.4	−32.24	−33.8	−27.2	−34.3

**Table 5:** Details of the −3 dB bandwidth and secondary lobe level from the X-Z PSF in the 22–26 GHz range.

IX	Cos q	GAA	GAA+Radome	GAA+HWradome	GAA+MTR
−3 dB PSF BW (m)	0.0143	0.0159	0.0155	0.0151	0.0162
PSF SLL (dB)	−35.6	−38.2	−33.8	−33.0	−31.8

## Data Availability

The original data presented in the study are openly available in “Dataset of Radomizing an Antenna for an SAR-Based ETA Radar System”, Mendeley Data, V2, https://doi.org/10.17632/5899xd56nd.2.
